# The Mechanisms Underlying the Protective Action of Selenium Nanoparticles against Ischemia/Reoxygenation Are Mediated by the Activation of the Ca^2+^ Signaling System of Astrocytes and Reactive Astrogliosis

**DOI:** 10.3390/ijms222312825

**Published:** 2021-11-26

**Authors:** Elena G. Varlamova, Egor A. Turovsky, Valentina A. Babenko, Egor Y. Plotnikov

**Affiliations:** 1 Federal Research Center “Pushchino Scientific Center for Biological Research, Russian Academy of Sciences”, Institute of Cell Biophysics of the Russian Academy of Sciences, 142290 Pushchino, Russia; 2 A.N. Belozersky Institute of Physico-Chemical Biology, Lomonosov Moscow State University, 119992 Moscow, Russia; nucleus-90@yandex.ru (V.A.B.); plotnikov@belozersky.msu.ru (E.Y.P.); 3 V.I. Kulakov National Medical Research Center of Obstetrics, Gynecology and Perinatology, 117997 Moscow, Russia

**Keywords:** oxygen-glucose deprivation, selenium nanoparticles, neurons, astrocytes, gene expression, reactive astrogliosis, calcium signaling, neuron-glia interactions, cytoprotection

## Abstract

In recent years, much attention has been paid to the study of the therapeutic effect of the microelement selenium, its compounds, especially selenium nanoparticles, with a large number of works devoted to their anticancer effects. Studies proving the neuroprotective properties of selenium nanoparticles in various neurodegenerative diseases began to appear only in the last 5 years. Nevertheless, the mechanisms of the neuroprotective action of selenium nanoparticles under conditions of ischemia and reoxygenation remain unexplored, especially for intracellular Ca^2+^ signaling and neuroglial interactions. This work is devoted to the study of the cytoprotective mechanisms of selenium nanoparticles in the neuroglial networks of the cerebral cortex under conditions of ischemia/reoxygenation. It was shown for the first time that selenium nanoparticles dose-dependently induce the generation of Ca^2+^ signals selectively in astrocytes obtained from different parts of the brain. The generation of these Ca^2+^ signals by astrocytes occurs through the release of Ca^2+^ ions from the endoplasmic reticulum through the IP3 receptor upon activation of the phosphoinositide signaling pathway. An increase in the concentration of cytosolic Ca^2+^ in astrocytes leads to the opening of connexin Cx43 hemichannels and the release of ATP and lactate into the extracellular medium, which trigger paracrine activation of the astrocytic network through purinergic receptors. Incubation of cerebral cortex cells with selenium nanoparticles suppresses ischemia-induced increase in cytosolic Ca^2+^ and necrotic cell death. Activation of A2 reactive astrocytes exclusively after ischemia/reoxygenation, a decrease in the expression level of a number of proapoptotic and proinflammatory genes, an increase in lactate release by astrocytes, and suppression of the hyperexcitation of neuronal networks formed the basis of the cytoprotective effect of selenium nanoparticles in our studies.

## 1. Introduction

Ischemic brain damage includes a cascade of signaling and metabolic events leading to the induction of necrotic and apoptotic processes. In this case, the following processes are observed: a decrease in the partial pressure of oxygen, the supply of tissue with glucose and other nutrients, an increase in the concentration of extracellular glutamate, an increase in cytosolic calcium ([Ca^2+^]_i_) in neurons and astrocytes, an increase in the production of ROS, etc. [1,2]. The search for new neuroprotective compounds with antioxidant and anti-inflammatory properties requires an interdisciplinary approach and remains an urgent task of modern sciences. In this vein, the microelement selenium (Se), discovered by Berzelius back in 1817, is of great interest. Today, it remains not fully understood and does not cease to amaze with the variety of its functions. The uniqueness of Se, first of all, lies in the fact that it is included not only in organic and inorganic compounds but also is a key component of the amino acid selenocysteine in selenoproteins, found in all domains of life. Being a 21 amino acid in the universal genetic code, selenocysteine is encoded by one of three translation stop codons; the presence of specific cis- and trans-active factors is necessary for its recognition as a selenocysteine codon. Of all the trace elements of the periodic table, only five (iron, molybdenum, manganese, zinc, and selenium) are part of the enzymes, but Se is the only trace element, which is the most important component of enzymes belonging to different classes and families: oxidoreductase, deiodinase, synthetase, etc.

The brain tissue is extremely sensitive to Se deficiency [3]. It has been shown that this microelement, acting through the activation of selenoproteins in the brain, is involved in the mechanisms of memory formation, motor, and cognitive activity [4]. Disorders of selenoprotein expression and Se metabolism in the brain are associated with a number of neurodegenerative diseases—epilepsy, Alzheimer’s disease, Parkinson’s disease, etc. [5]. At the same time, it was shown that exogenous Se and its compounds are able to alleviate the course of these diseases, primarily through suppression of oxidative stress, modulation of Ca^2+^ homeostasis, and mitochondrial biogenesis, contributing to the maintenance of the energy balance of brain cells and their survival in the penumbra zone [6,7,8].

Despite the existing methods of preventing or alleviating the consequences of ischemic cerebral stroke, a large problem of the blood-brain barrier remains, and most cerebroprotectors poorly penetrate it. This is typical for Se compounds. In this sense, selenium nanoparticles (SeNPs) are of interest as an effective neuroprotector capable of easily crossing the blood-brain barrier. In addition, SeNPs have powerful antioxidant properties against a background of reduced cytotoxicity compared to other Se-containing compounds [9,10]. There are studies demonstrating the neuroprotective properties of SeNPs in Alzheimer’s disease, which inhibit the aggregation of beta-amyloid and its toxicity [11,12]. In epilepsy, SeNPs increased the expression of GPx, SOD2, NRF-2, and GABA against the background of a decrease in the level of ROS, NO, proinflammatory cytokines, and extracellular glutamate [13]. In addition, the application of SeNPs in Parkinson’s and Huntington’s diseases led to an increase in the antioxidant capacity of brain cells, an increase in the expression of glutathione peroxidases, and suppression of apoptotic processes [14,15].

Based on the foregoing, most studies demonstrate the antioxidant effect of SeNPs during neurodegeneration and, as a result, suppression of apoptosis. Ischemia and reperfusion are associated with a pathological increase in ROS production, a global increase in the concentration of [Ca^2+^]_i_, and cell death [16]. The mechanisms of cell death are closely related and regulated by changes in [Ca^2+^]_i_. Our results obtained on the human glioblastoma cell line A-172 showed the activation of the [Ca^2+^]_i_ signaling system of cells in response to the addition of selenium nanoparticles [17]. This work focuses on the mechanisms of calcium signaling in brain cells under the action of SeNPs and their neuroprotective properties during ischemia/reoxygenation.

## 2. Results

### 2.1. SeNPs Dose-Dependently Induce the Generation of Ca^2+^ Signals in Astrocytes, but Not in Neurons of Various Parts of the Brain

**Figure 1:**
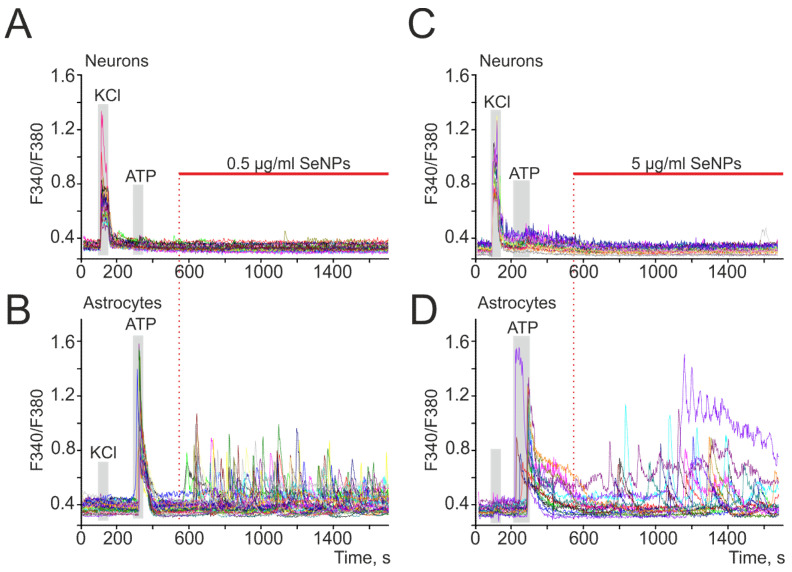
SeNPs dose-dependently induce the generation of Ca^2+^ signals in astrocytes, but not in neurons of various parts of the brain. (**A**–**D**) Generation of Ca^2+^ signals in astrocytes (**B**,**D**) in response to the application of 0.5 and 5 µg/mL SeNPs, respectively; (**A**,**C**) absence of Ca^2+^ signals in neurons. Ca^2+^ signals in neurons and astrocytes in response to SeNPs application in one experiment are presented here.

**Figure 2:**
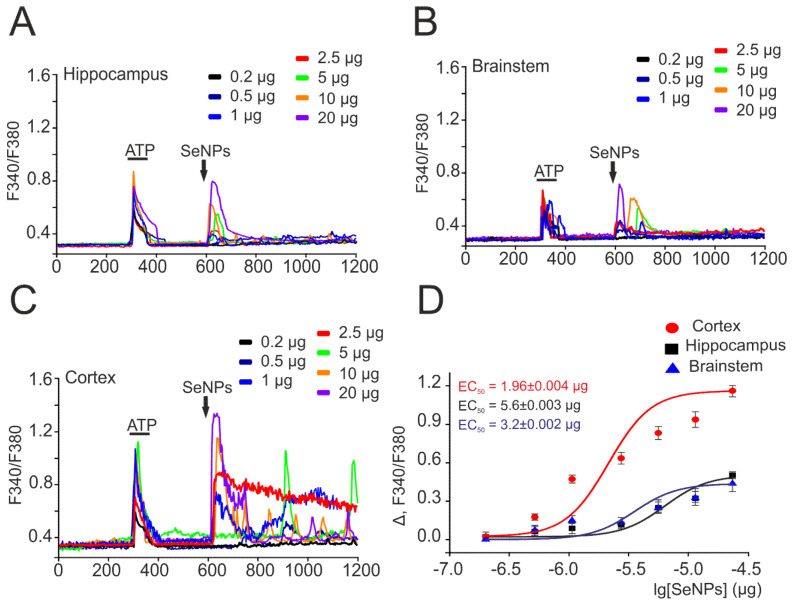
Dose-dependent change in the amplitude of Ca^2+^ signals in response to the application of SeNPs in astrocytes obtained from different parts of the mouse brain. (**A**–**C**) Ca^2+^ signals of astrocytes obtained from the hippocampus (**A**), brainstem (**B**), and cortex (**C**) for application of various concentrations of SeNPs (0.2–20 µg/mL). (**D**) Dependence of the amplitude of Ca^2+^ responses of astrocytes from different parts of the brain on the growth of SeNPs concentration and its approximation by a sigmoid function. Neuroglial cultures of different parts of the brain were obtained from one mouse and cultured under the same conditions. For (**A**–**C**), Ca^2+^ signals averaged over several tens of astrocytes are presented. To plot dose dependences, we used the results of Ca^2+^-dynamic measurements on three independent cell cultures.

The addition of 0.5 µg/mL SeNPs to neuroglial cultures of the mouse cerebral cortex did not induce Ca^2+^ responses in neurons (Figure 1A), while Ca^2+^ signals were recorded in astrocytes mainly in the form of oscillations (Figure 2B). An increase in the concentration of SeNPs to 5 µg/mL also did not induce the generation of Ca^2+^ signals in neurons of the cerebral cortex (Figure 1C), but an increase in the amplitude of Ca^2+^ responses and the appearance of signals at an increased base level of [Ca^2+^]_i_ was observed in astrocytes (Figure 1D).

To confirm the selectivity of the action of SeNPs on astrocytes, neuroglial cultures were obtained from three parts of the mouse brain—the hippocampus, brainstem, and cortex. Figure 2 shows the results of this series of experiments, according to which the generation of Ca^2+^ signals exclusively in astrocytes in all studied brain regions was observed upon application of various concentrations of SeNPs. Interestingly, upon application of 0.5 µg/mL SeNPs, an increase in [Ca^2+^]_i_ signals was observed only in astrocytes obtained from the cerebral cortex (Figure 2C,D), while cells from the hippocampus (Figure 2A) and brainstem (Figure 2B) began to respond only to the addition of 1 µg/mL SeNPs and above (Figure 2D). In addition, the amplitudes of Ca^2+^ signals in response to the addition of SeNPs were higher in astrocytes of the cerebral cortex (Figure 2C), while astrocytes of the hippocampus and brainstem responded mainly with transient Ca^2+^ signals (Figure 2A,B). The approximation of the amplitudes of Ca^2+^ signals by the sigmoid function in astrocytes from different parts of the brain showed different EC50 values for the activation of the Ca^2+^ signal system of cells. For cortical astrocytes, the EC50 value was 1.96 ± 0.004 μg/mL (Figure 2D, red curve), while for astrocytes of the hippocampus and brainstem, this value was 5.6 ± 0.003 μg/mL and 3.2 ± 0.002 μg/mL (Figure 2D, black and blue curves).

Thus, the application of SeNPs to neuroglial cultures of different parts of the brain causes the generation of Ca^2+^ signals exclusively in astrocytes, and the amplitude of these responses increases with an increase in the dose of SeNPs. Astrocytes of the cerebral cortex turned out to be the most sensitive to the activation of the Ca^2+^-signal system in response to the application of SeNPs, and it was decided to conduct further experiments to study the mechanisms of action of SeNPs on neuroglial cultures of the cerebral cortex.

### 2.2. SeNPs Induce Ca^2+^ Signals in Astrocytes through the Activation of the Phosphoinositide Signaling Cascade and the Mobilization of Ca^2+^ Ions from the Thapsigargin-Sensitive ER Pool

**Figure 3:**
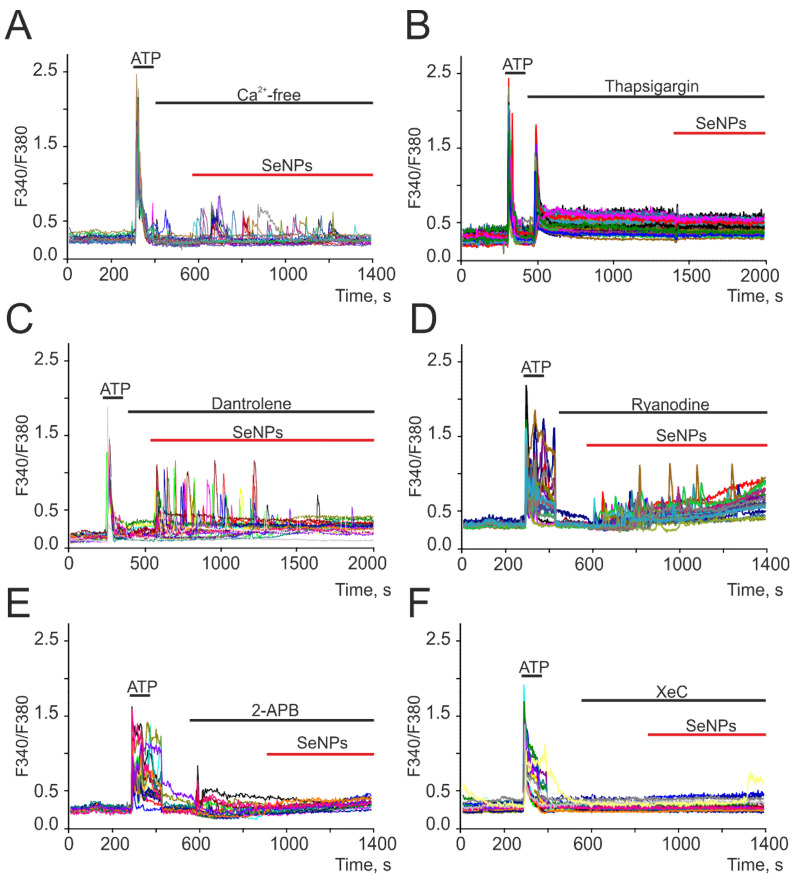
Generation of Ca^2+^ signals in cortical astrocytes in neuroglial culture in response to the application of 0.5 μg/mL SeNPs in calcium-free medium containing 0.5 mM EGTA (**A**), after depletion of ER with thapsigargin (**B**, 10 μM), in the presence of ryanodine receptor antagonists dantrolene (**C**, 30 μM) and ryanodine (**D**, 100 μM) and IP_3_R inhibitors 2-APB (**E**, 30 µM) and xestospongin C (**F**, XeC, 3 µM). Here, Ca^2+^ signals of astrocytes in one experiment are presented.

To generate Ca^2+^ signals, cells use the Ca^2+^ stored in the intracellular depot and the entrance of Ca^2+^ ions from the extracellular environment. Application of 0.5 μg/mL SeNPs in a calcium-free medium did not abolish the generation of Ca^2+^ responses by astrocytes (Figure 3A), but the amplitude and frequency of Ca^2+^ oscillations decreased. The emptying of the ER using thapsigargin completely blocked the generation of Ca^2+^ signals in cells in response to the addition of 0.5 μg/mL SeNPs (Figure 3B). The intracellular receptor channels responsible for the mobilization of [Ca^2+^]_i_ from the ER are ryanodine (RyR) and IP_3_-receptors (IP_3_R). RyR antagonists dantrolene (Figure 3C) and ryanodine (Figure 3D) did not affect the SeNPs-induced increase in [Ca^2+^]_i_. Blocking IP_3_R with 2-APB (Figure 3E) or xestospongin C (XeC, Figure 3F) completely stopped astrocyte responses to the addition of SeNPs.

Thus, the Ca^2+^ signals of astrocytes in response to the addition of SeNPs occurred due to the mobilization of Ca^2+^ ions from the thapsigargin-sensitive ER pool through IP_3_R, i.e., due to the activation of the phosphoinositide signaling cascade.

### 2.3. SeNPs Involve ATP-Release Mechanisms through the Activation of Connexin Hemichannels and Activation of P2-Purinoreceptors in the Astrocytes

**Figure 4:**
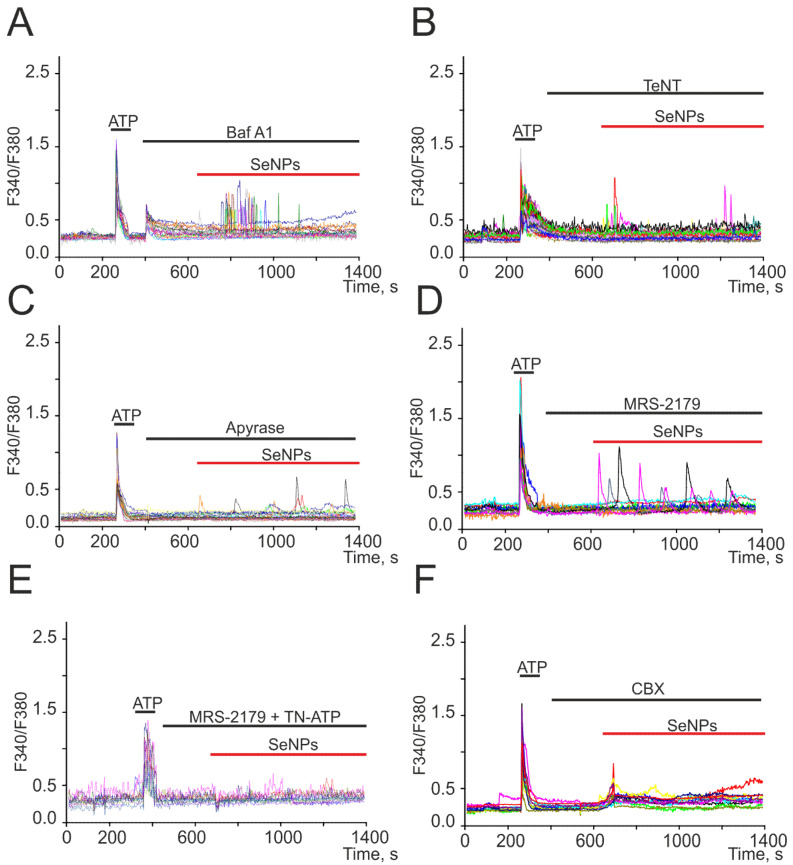
Suppression of Ca^2+^ signals in cortical astrocytes in response to the application of 0.5 µg/mL SeNPs in the presence of a vesicular secretion blocker bafilomycin A1 (**A**, Baf A1, 10 µM), tetanus toxin (**B**, TeNT, 100 µg/mL), an inhibitor of Ca^2+^-dependent vesicular fusion, apyrase (**C**, 30 U/mL), an enzyme that breaks down ATP, P2Y receptor antagonist MRS-2179 (**D**, 30 µM), combination MRS-2179 with P2X receptor antagonist TN-ATP (**E**, 10 µM), and connexin blocker carbenoxolone (**F**, CBX, 100 µM). Here, Ca^2+^ signals of cells in one experiment are presented.

We have previously shown on a human glioblastoma cell line (A-172) that the addition of SeNPs promotes the activation of secretion mechanisms [17]. In neuroglial networks, astrocytes are cells that secrete a number of gliotransmitters and regulate the activity of neurons [18,19,20]. Incubation of cerebral cortex astrocytes with bafilomycin A1 (Baf A1) resulted in inhibition of Ca^2+^ oscillations in response to the addition of SeNPs, and Ca^2+^ signals from cells appeared as single transients (Figure 4A) or were completely absent. A blocker of Ca^2+^-dependent vesicular secretion mechanisms, tetanus toxin (TeNT, Figure 4B), prevented the generation of Ca^2+^ signals in most astrocytes in response to the addition of 0.5 μg/mL SeNPs, and transient Ca^2+^ signals were recorded only in single cells.

For intercellular communication, astrocytes secrete ATP in response to external stimuli [18], and preincubation of cells with an ATP-degrading enzyme apyrase (Figure 4C) resulted in suppression of Ca^2+^ signals in response to the addition of 0.5 µg/mL SeNPs in most astrocytes. Only in single cells were low-amplitude Ca^2+^ signals recorded, which could be caused by the secretion of other gliotransmitters. In the remaining 30% of cells, ATP secretion activated P2X receptors, which was confirmed by the disappearance of Ca^2+^ signals in response to SeNPs after incubation of cerebral cortex cultures with MRS-2179 and a P2X receptor blocker (TN-ATP) simultaneously (Figure 4E). It is known that ATP release in nonexcitable cells [21], including astrocytes [20,22], may be due to the discovery of Cx43. Carbenoxolone, connexin blocker (CBX, Figure 4F), completely prevented the generation of Ca^2+^ oscillations in response to 0.5 µg/mL SeNPs application, but a number of cells retained low-amplitude transients due to the mobilization of Ca^2+^ ions from the ER.

Thus, the generation of Ca^2+^ signals in the astrocytic network in response to the application of SeNPs involves the mechanisms of vesicular Ca^2+^-dependent ATP secretion through connexin (Cx43) channels, which activates the purinergic receptors of nearby astrocytes.

### 2.4. SeNPs Protect Cortical Cells from OGD-Induced Necrosis through Suppression of Ca^2+^ Signals, Decreased Respiration, and Increased Lactate Release by Astrocytes

**Figure 5:**
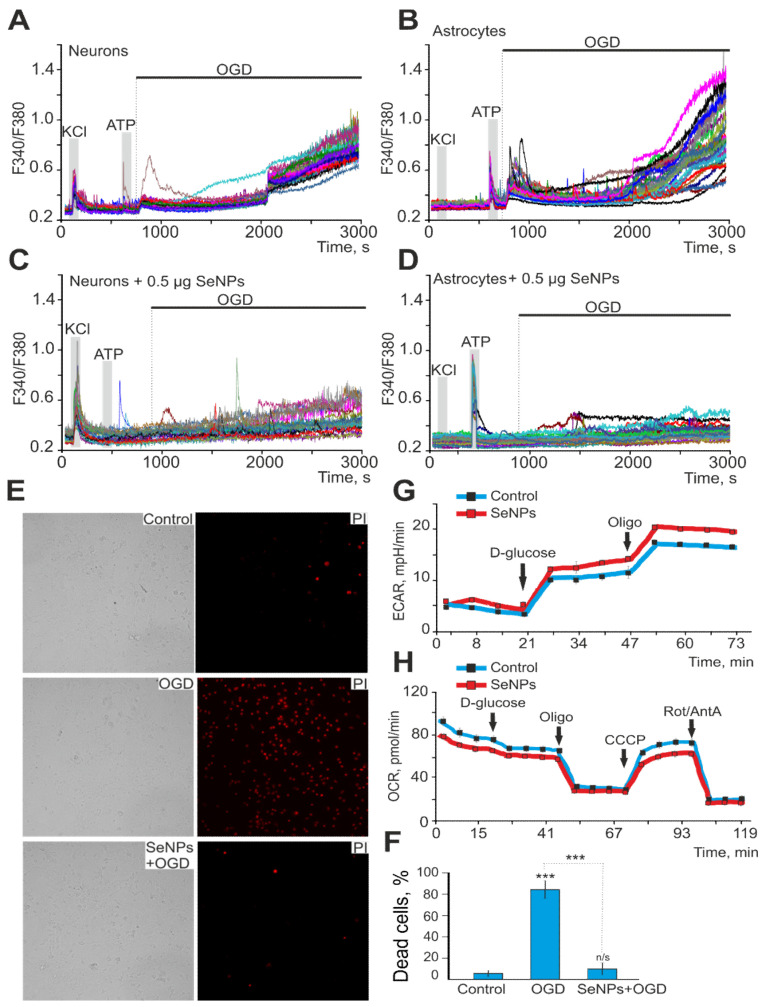
Protective effect of 0.5 μg/mL SeNPs from OGD-induced [Ca^2+^]_i_ increase and necrosis. (**A**,**B**) Ca^2+^-signals of neurons (**A**) and astrocytes (**B**) during 40 min OGD. (**C**,**D**) Ca^2+^-signals of neurons (**C**) and astrocytes (**D**) during 40 min OGD after 24 h incubation with 0.5 μg/mL SeNPs. (**E**) Images of cortical cell culture in transmitted light and propidium iodide fluorescence detection channel in control (without OGD), after 40 min OGD (OGD) and 24 h treatment with 0.5 μg/mL SeNPs (SeNPs + OGD). The white dots represent the PI-stained nuclei of necrotic cells. (**F**) Average number of PI-stained cells that died in control due to OGD-induced necrosis in the absence of SeNPs (OGD) and after 24 h incubation with SeNPs (SeNPs+OGD) (% ± SE). Short-term applications of 35 mM of KCl and 10 µm of ATP were used to detect neurons and astrocytes, respectively. Statistical significance was assessed using paired *t*-test. n/s—data not significant (*p* > 0.05), *** *p* < 0.001. (**G**,**H**) Effect of 2.5 μg of SeNPs on the rate of acidification of the extracellular medium ECAR (**G**) and the rate of oxygen consumption OCR (**H**) in astrocytes after the application of 10 mM D-glucose, 4.5 μM of oligomycin, and a mixture of 2.5 μM of rotenone and 4 μM of antimycin A.

Ischemia-like conditions (OGD) for 40 min cause the generation of biphasic Ca^2+^ signals in neurons (Figure 5A) and astrocytes (Figure 5B), which correlates with necrotic cell death (Figure 5F, OGD), recorded by the appearance of fluorescence PI after 40 min OGD. In control cell cultures of the mouse cerebral cortex, 8 ± 3% of necrotic cells can be recorded in vitro by Day 10 (Figure 5E,F), but after 40 min of OGD, necrosis is recorded in 83 ± 11% of cells. It is known that SeNPs have pleiotropic effects [23] and, on the one hand, activate apoptotic processes in cancer cells [17,24], but on the other hand, they are capable of exerting neuroprotective effects [25]. In addition, we have shown that the protective effects of the anti-inflammatory cytokine, interleukin-10, and antioxidants increase in proportion to the incubation time and are largely realized through the protection of astrocytes [20,22]. Incubation of cerebral cortex cell cultures with 0.5 μg/mL SeNPs for 24 h leads to suppression of both phases of the [Ca^2+^]_I_, OGD-induced increase in neurons (Figure 5C) and astrocytes (Figure 5D), which leads to a decrease in necrotic cell death up to 13 ± 11% (Figure 5E,F).

Measurements of respiration and lactate release by cortical astrocytes immediately after the application of SeNPs showed that the rate of oxygen consumption decreases (Figure 5G), while extracellular acidification, mediated primarily by the release of lactate, on the contrary, increases (Figure 5H), which may be associated with protective effects of nanoparticles on neuroglial networks in OGD. Each curve in Figure 5G describes the changes in ECAR in cells during the assay time under baseline (no glucose in the measurement medium), in the presence of D-glucose, and after oligomycin application. As a result of ECAR measurements, 10 min after the application of 2.5 μg of SeNPs to the medium with astrocytes, the ECAR values were higher both in the state of inactivated glycolysis after glucose application and under conditions when astrocytes make the most glycolysis for energy production (after adding an ATP synthase inhibitor oligomycin) compared with the corresponding values for native astrocytes (Figure 5G). On the other hand, an increase in the rate of acidification of the extracellular environment may indicate not only an increase in glycose but also an increase in the release of lactate from the cell under the action of SeNPs.

At the same time, when analyzing the effect of SeNPs on astrocyte respiration, a decrease in the rate of oxygen consumption (OCR) was revealed in comparison with native astrocytes (Figure 5H). Thus, respiration rate was decreased in SeNP-treated astrocytes under conditions of basal respiration after the application of D-glucose and after the application of CCCP; that is, under conditions of maximally stimulated (uncoupled) respiration. Thus, the presence of SeNPs in the medium leads to a decrease in the rate of oxygen consumption by astrocytes and an increase in the acidification of the extracellular medium. It can be assumed that SeNPs induce a shift in metabolism in astrocytes towards glycolysis as opposed to respiration. As a result, the production of lactate is significantly increased, and its release in large quantities into the extracellular environment is significantly increased.

Thus, SeNPs protect neuroglial networks from ischemia in vitro through suppression of the global increase in [Ca^2+^]_i_, decreased oxygen consumption rate, and increased lactate release, which results in suppression of necrotic cell death.

### 2.5. Neuroprotective Effects of SeNPs in Ischemia/Reoxygenation Involve Altered Expression of Genes Encoding Kinases, Neurotrophins, and Transcription Factors

**Figure 6:**
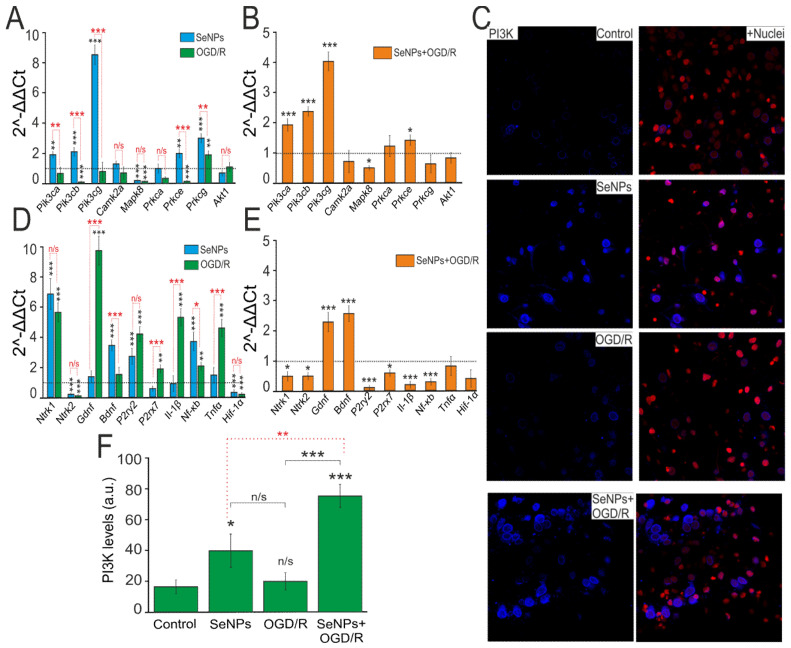
Effect of OGD (40 min) with 24 h reoxygenation (OGD/R), 24 h incubation of cortical cells with 0.5 μg/mL SeNPs (SeNPs), and 24 h incubation with 0.5 μg/mL SeNPs with OGD/R (SeNPs+OGD/R) on the level expression of genes encoding signal kinases (**A**,**B**), receptors, neurotrofins, and proinflammatory factors (**D**,**E**). Dashed line for (**A**,**D**) indicates level of gene expression in controls (without treatments). Dashed line for (**B**,**E**) indicates level of gene expression in OGD/R cortical cultures. Statistical significance was assessed using paired *t*-test. n/s—data not significant (*p* > 0.05), * *p* < 0.05, ** *p* < 0.01, and *** *p* < 0.001. (**C**) Immunostaining of PI3K in cells of the cerebral cortex in the control, after 24 h of incubation with 0.5 μg/mL SeNPs, and after OGD/R and OGD/R after 24 h of incubation with SeNPs+Nuclei—Draq5 nuclei staining. (**F**) Intensity levels of PI3K were determined by confocal imaging. We analyzed individual cells that had fluorescence of secondary antibodies. The quantitative data reflecting the level of PI3K expression are presented as fluorescence intensity values in summary bar charts (mean +/− SEM). The values were averaged by 200 cells for each column. The results obtained after immunostaining agree well with the data of fluorescence presented in (**C**). Each value is the mean ± SE (*n* ≥ 3, *p* < 0.05). Statistical significance was assessed using paired *t*-test. Comparison with control, *** *p*-level < 0.001, * *p*-level < 0.05, n/s—data not significant (*p* > 0.05).

We have previously shown that application of SeNPs to A-172 glioblastoma cells and other cancer lines leads to a significant change of a number of key proapoptotic and proinflammatory genes [17,24]. Incubation of cortical cultures with 0.5 μg/mL SeNPs for 24 h led to an increase in the basic expression of genes encoding PI3K (*Pik3*) and PKC (*Prkc*) subunits by 2–8 times and 2–3.7 times, respectively (Figure 6A, blue columns). On the contrary, after 40 min of OGD and 24 h of reoxygenation (OGD/R), there was a tendency to suppress the expression of genes encoding these kinases (Figure 6A, green bars). Addition of 0.5 μg/mL SeNPs for 24 h and subsequent exposure to OGD/R maintained a trend towards an increase in the expression of genes encoding PI3K (Figure 6B), while for PKC, an increase in the expression of only one subunit (*Prkce*) was observed.

The results on changes in the expression of genes encoding PI3K subunits were confirmed by immunocytochemical staining of cells with specific antibodies against PI3K (Figure 6C). After incubation of cells with 0.5 μg/mL SeNPs during 24 h, there was an increase in the PI3K protein content inside the cells (Figure 6C,F, SeNPs), while under OGD/R conditions, no significant change in PI3K expression was observed (Figure 6C,F, OGD/R). Preincubation of cortical cultures with 0.5 μg/mL SeNPs and subsequent modeling of OGD/R led to a twofold increase in the expression of the PI3K protein inside cells (Figure 6C,F, SeNPs+OGD/R), compared with the action of the SeNPs themselves and a threefold increase in comparison with control and OGD/R effect.

In addition to altering the expression of genes encoding kinases, incubation of cells with 0.5 μg/mL SeNPs for 24 h led to an increase in the expression level of genes encoding TrkA (*Ntrk1*), BDNF, P2Y receptors (*P2ry2*), and *Nf-κB* by 6, 3.8, 3.5, and 4 times, respectively (Figure 6D, blue bars). After OGD/R, there was an increase in the expression of TrkA (*Ntrk1*), P2Y receptors (*P2ry2*), P2X receptors (*P2rx7*), *Il-1β*, *Nf-κB*, and *Tnfα* by 2.5–10 times (Figure 6D, green bars). Both when cells were incubated with SeNPs and after OGD/R, the expression of TrkB (*Ntrk2*) and *Hif-1α* was suppressed. Preincubation of cells with 0.5 μg/mL SeNPs and subsequent OGD/R led to a decrease in the expression level of most of the studied genes, *Ntrk1*, *Ntrk2*, *P2ry2*, *P2rx7*, *Il-1β*, and *Nf-κB,* against the background of increased expression of genes encoding neurotrophins BDNF and GDNF (Figure 6E).

Thus, the preliminary incubation of cortical cultures with SeNPs generally promotes a change in the expression of a number of key neuroprotective genes aimed at increasing the basic viability of cells.

### 2.6. Reactivation of Astrocytes under the Action of SeNPs as an Underlying Mechanism of Neuroprotection against OGD/Reoxygenation

**Figure 7:**
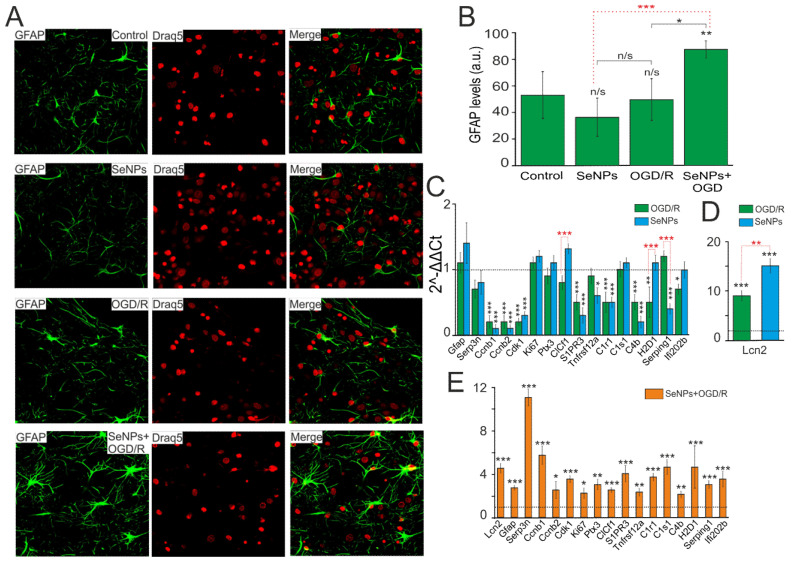
Effect of OGD/R, 24 h incubation with 0.5 μg/mL SeNPs and OGD/R after 24 h incubation with 0.5 μg/mL SeNPs on astrocyte reactivity. (**A**) Immunocytochemical staining of cortical cell culture with astrocytic marker, antibodies against glial fibrillary acidic protein (GFAP). The nuclei of all cells stained with Draq5 are shown in blue. (**B**) Intensity levels of GFAP were determined by confocal imaging. We analyzed individual cells that had fluorescence of secondary antibodies. The quantitative data reflecting the level of GFAP expression are presented as fluorescence intensity values in summary bar charts (mean +/− SEM). The values were averaged by 150–200 cells for each column. The results obtained after immunostaining agree well with the data of fluorescence presented in (**A**). Each value is the mean ± SE (*n* ≥ 3, *p* < 0.05). Statistical significance was assessed using paired *t*-test. Comparison with control, n/s—data not significant (*p* > 0.05), ** *p* < 0.01, *** *p* < 0.001. (**C**,**D**) SeNPs and OGD/R-induced changes in the expression of genes encoding marker proteins of astrocyte reactivity. Dashed line for (**C**,**D**) indicates level of gene expression in controls (without treatments). (**E**) effect of 24 h incubation of cells with 0.5 μg/mL SeNPs on OGD/R-induced astrocyte reactivity. Dashed line for (**E**) indicates level of gene expression in OGD/R cortical cultures. Statistical significance was assessed using paired *t*-test. n/s—data not significant (*p* > 0.05), * *p* < 0.05, ** *p* < 0.01, and *** *p* < 0.001.

It is known that in response to various pathological stimuli in astrocytes, changes in the regulation of transcription can occur, as well as biochemical, morphological, metabolic, and physiological remodeling, characterized by the general name “reactive astrogliosis”. One of the well-known markers of astrocyte reactivation is an increase in glial fibrillary acid protein (GFAP). Incubation of cerebral cortex cells with 0.5 μg/mL SeNPs and higher, as well as OGD/R, does not significantly change the level of GFAP inside astrocytes (Figure 7A,B), while preliminary incubation with SeNPs and subsequent OGD/R leads to an increase in the level of intracellular GFAP in astrocytes, which may indicate activation of reactivity.

To more reliably establish the presence or absence of reactive astrocytes in the studied samples under conditions of oxygen-glucose deprivation with and without pretreatment of cells with SeNPs, a complex comparison of the mRNA expression patterns of genes of various reactivity markers, characterized earlier [26,27,28,29], was carried out.

According to the results of real-time PCR, we can conclude, firstly, that in the neuroglial culture of the mouse cerebral cortex under OGD/R conditions (Figure 7C, green bars) and after 24 h incubation with SeNPs (Figure 7C, blue bars), no increase in the expression of key markers of astrocyte reactivation was observed, except for the gene encoding the secreted lipophilic protein *Lcn2* (Figure 7D). The expression of *Lcn2* mRNA increased 9 and 15 times during OGD and incubation with SeNPs, respectively; however, despite the fact that this protein, according to some data [30,31], is involved in reactive astrocyte gliosis, a significant decrease in the expression of other markers may indicate about the absence of reactive astrogliosis in the studied samples.

Second, when analyzing the expression of astrogliosis markers in cell samples, after OGD/R with preliminary 24 h treatment with SeNPs, an opposite picture of their expression patterns was observed. This may indicate the presence of signs of astrocyte reactivity (Figure 7E). Thus, compared with the expression of these genes after OGD and without pretreatment with SeNP, the cells showed an increase in expression of the widely used marker of reactive astrocytes *GFAP* by 2.7 times, *Lcn2* (by 4.5 times), and *Serpina3n*, a secreted peptidase inhibitor (by 11 times), the expression of which is induced by inflammation and nerve damage [32,33]. Activation of proliferation genes was also observed, including late-phase cyclins b1 and b2 (*Ccnb1* and *Ccnb2*) by 5.7 and 2.5 times, respectively, *Cdk1* (by 3.5 times), and proliferation marker *Ki67* (by 2.2 times). Of great interest is the fact that many signaling receptors are very strongly activated in reactive astrocytes, including the receptors opsonin *Ptx3*, sphingosine-1-phosphate receptor 3 (*S1PR3*), and TNF receptor superfamily member 12A (*TNFRSF12A*), which was also demonstrated in our work.

It is known that the nature of reactive astrogliosis differs depending on the stimulus. Previously, it was shown that the characteristic markers of astrogliosis caused by middle cerebral artery occlusion (MCAO) and neuroinflammation (LPS) are the MHC class I molecules, *H2*-*D1* and *Serping1*, a *C1q* inhibitor that is a critical regulator of complement activity [34]. In addition, there was an increase in the expression of the genes *C1r* (3.7 times), *C1*s (4.6 times), and *C4* (2.1 times), which are activated, as a rule, in the initial part of the classical complement cascade. According to the results shown in Figure 7, the expression of mRNA *H2-D1* increased 4.6 times and *Serping1* 3 times, compared with the control. Consequently, the preliminary 24 h treatment of the neuroglial culture of the mouse cerebral cortex with SeNPs and the subsequent OGD/R result in the reactivation of astrocytes, predominantly of the A2 phenotype, with neuroprotective properties. The effect on cells only of SeNPs, or under OGD/R conditions without preliminary incubation with SeNPs, such effects were not observed.

**Figure 8:**
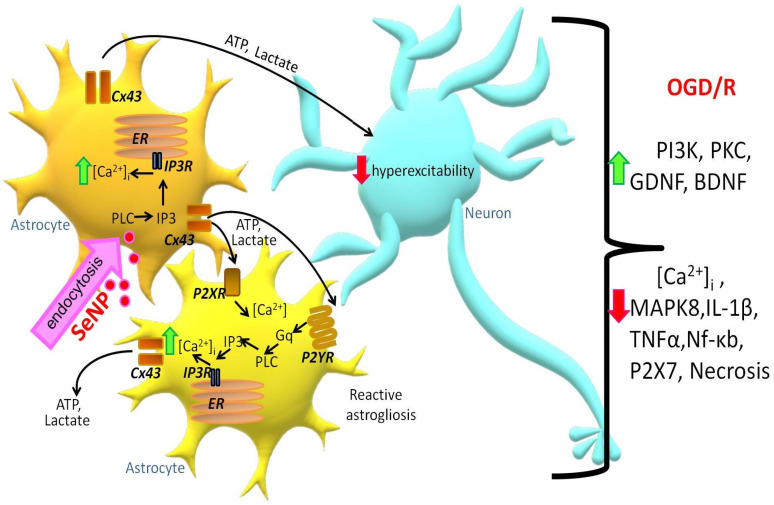
Signaling scheme in neuroglial cell culture under the action of SeNPs. Designations: ER—endoplasmic reticulum; Cx43—connexin hemichannels; IP_3_R—inositol trisphosphate receptor; IP_3_—inositol trisphosphate; PLC—phospholipase C; ATP—adenosine triphosphate; P2YR and P2XR—Y and X types of purinoreceptors; Gq—Gq alpha subunit of G-protein-coupled receptors.

Thus, application of SeNPs induces the generation of Ca^2+^ signals in astrocytes through the mobilization of Ca^2+^ ions from the ER via IP_3_R upon activation of the phosphoinositide signaling pathway. An increase in [Ca^2+^]_i_ in the astrocyte causes Ca^2+^-dependent ATP secretion and lactate release through the Cx43 connexin chemical channels (Figure 8). As a result, paracrine activation of the entire astrocyte network and suppression of OGD-induced hyperexcitation of neurons occurs. Prolonged incubation of cerebral cortex cells with SeNPs leads to inhibition of OGD-induced global increase in [Ca^2+^]_i_ and necrotic death through activation of reactive astrogliosis and expression of genes encoding cytoprotective proteins.

## 3. Discussion

The trace element Se has a pleiotropic effect and has a high therapeutic potential for the treatment of various diseases, and its compounds and nanoparticles are of great interest in biotherapy and nanomedicine [35]. During ischemia/reoxygenation, increased oxidative stress, activation of glutamate receptors, Ca^2+^ overload of mitochondria, and activation of inflammatory processes due to increased release of proinflammatory factors by glial cells occur in the brain, which leads to damage and death of neurons [5,36]. In recent decades, the role of nanoparticles in neurological diseases has been actively studied, since neurons are especially vulnerable to damage caused by oxidative stress due to high oxygen consumption, the presence of a large amount of polyunsaturated fatty acids, and a low level of expression of antioxidant enzymes [37,38]. The other types of brain-resident cells such as microglia and vascular endothelial cells are also targets for the treatment of neurodegeneration with selenium nanoparticles and selenium quantum dots [39,40]. 

Stimulation of apoptosis with large doses of SeNPs may have a positive effect in the case of oncological diseases, since SeNPs trigger a metabolic transition from uncontrolled cell death, necrosis, to a controlled pathway of cell death—apoptosis. This could have potential therapeutic value. Examples of the use of SeNPs as an anticancer agent are widely known [10,41]. In healthy tissue, the use of high concentrations of nanoparticles seems to be dangerous; however, as our studies have shown, low concentrations of SeNPs are capable of causing cytoprotective effects through the activation of the Ca^2+^ signaling system of astrocytes. Interestingly, astrocytes obtained from different parts of the brain exhibit different sensitivity to SeNPs, which can be explained by different levels of expression of receptors, channels, and Ca^2+^-transport systems [42,43].

At the same time, in normal brain tissue during ischemia/reoxygenation, there is a need to suppress the processes of necrosis and apoptosis. Our experiments showed that after incubation with SeNPs, not only complete inhibition of OGD/R-induced necrosis occurred but also an increase in the expression of PI3K and PKC, the role of which in suppressing apoptosis of brain cells is well known [44,45,46,47,48]. Our experiments have shown that incubation with SeNPs leads to suppression of *IL-1β* and *Nf-κb* expression after OGD/R exposure. Other authors, using an epilepsy model, found that SeNPs not only suppress oxidative stress but also inflammation by inhibiting *NF-κB* [13,46]. In a mouse model of stroke, it was found that SeNP, entering the brain cells by endocytosis mediated by transferrin receptors, also inhibits the inflammatory response and increases the survival of hippocampal neurons [49].

It is well known that disorders of Ca^2+^ homeostasis of brain cells accompany not only neurodegenerative diseases [50] but also cause the induction of necrosis and apoptosis during ischemia/reoxygenation [5,51]. In our experiments, incubation of neuroglial cultures of the cerebral cortex with SeNPs resulted in complete inhibition of both phases of the OGD-induced increase in [Ca^2+^]_i,_ which correlated with the suppression of cell death. There are studies that SeNPs are able to act through the regulation of Ca^2+^ homeostasis in nerve cells through increased expression of parvalbumin [52,53] and prevent cell death when exposed to excitotoxic doses of glutamate. In addition, ER-stress and increased cell death occur during ischemia/reoxygenation, while SeNPs are able to significantly suppress ER-stress, which may be another mechanism of their neuroprotective action [54,55].

Astrocytes are more resistant to death due to ischemia [56] and are able to suppress neuronal hyperexcitation for some time by increasing the secretion of ATP, gliotransmitters, and lactate release, preventing their death [20,57,58,59]. Application of SeNPs protects cortical cells from OGD/R-induced death by activating the paracrine secretion of ATP and lactate by astrocytes. Moreover, the key protein for signaling SeNPs, Cx43, is expressed exclusively in astrocytes, which may explain the absence of Ca^2+^ signals in neurons [42]. Application of SeNPs and subsequent OGD/R conditions lead to the induction of reactive astrogliosis, which likely promotes astrocyte phenotype modification and protects the neuroglial network from ischemic injury. Despite the fact that recently the classification of astrocytes into two groups (A1 and A2), according to their functional phenotype, is rather arbitrary, there are still some differences in the expression profile of a number of genes [60]. It is known that reactive astrocytes A1 have lost many of the characteristic functions of astrocytes, including promoting the survival and growth of neurons, the formation and functioning of synapses, and the ability to phagocytose synapses and myelin debris. At the same time, A2, which are induced by ischemia [60], strongly promote neuronal survival and tissue repair [61,62,63,64].

Therefore, there is some specificity in the expression of a number of markers of astrogliosis that characterize the functional phenotype of the reactive astrocyte. So, we have established an increase in the expression of genes interferon activated gene 202B *(Ifi202b*), which has anti-inflammatory activity; cardiotrophin-like cytokine factor 1 (*Clsf1*), a neurotropic cytokine that plays a decisive role in the development of neurons; and pentraxin-related protein (*Ptx3*), which plays an important role in the regulation of inflammatory reactions and increases expression of proliferation genes. These data may indicate the activation of neuroprotective A2 astrocytes.

## 4. Materials and Methods

Experimental protocols were approved by the Bioethics Committee of the Institute of Cell Biophysics. Experiments were carried out according to Act708n (23 August 2010) of the Russian Federation National Ministry of Public Health, which states the rules of laboratory practice for the care and use of laboratory animals, and the Council Directive 2010/63 EU of the European Parliament on the protection of animals used for scientific purposes. 

### 4.1. Preparation of Mixed Neuroglial Cell Cultures

Cell cultures from hippocampus, cortex, and brainstem were prepared as described in detail previously [5,65,66]. Briefly, 0–1-day-old pups were euthanized and decapitated. The extracted tissue was washed with Mg^2+^- and Ca^2+^-free Versene solution and minced with scissors. Then, the tissue fragments were digested with 1% trypsin solution for 10 min at 37 °C and washed two times with cold Neurobasal-A medium. Trypsinized tissue was gently triturated with a pipette, and the debris was then carefully removed with a pipette tip. The obtained cell suspension was seeded on polyethyleneimine-coated glass coverslips and grown for 10 days in vitro in cell culture medium composed of Neurobasal-A medium, supplement B-27 (2%), and 0.5 mM glutamine. 

The drugs were added into the culture medium under sterile conditions in the case of experiments with 24 h preincubation with SeNPs. Then, the cell cultures were washed after the preincubation with Hank’s balanced salt solution and used in experiments. 

### 4.2. Primary Astrocytes Culture

Astroglial cell cultures were isolated from the brains of 1–2-day-old rats according to the modified McCarthy and de Vellis protocol [67]. The brains were extracted, the cerebral cortex was separated, the meninges were removed, and tissue was ground and incubated in 0.05% trypsin-EDTA solution at 37 °C for 30 min. After enzymatic digestion, the tissues were washed twice in PBS and then dissociated by glass Pasteur pipette in a culture medium consisting of DMEM (PanEco, Russia), 1 g/L D-glucose, and 10% FBS (Biosera, Kansas City, MO, USA), with the addition of 2 mM glutamine (PanEco, Russia). The suspension of cells was transferred on ventilated culture vials (Costar, Washington, DC, USA) precoated with poly-D-lysine (10 mcg/mL). The cells were cultivated at 37 °C and 5% CO_2_. After 5–6 days, the cultures were subjected to vibration on an orbital shaker at 200 rpm for 16 h to detach and remove microglia. After 10 to 20 days, in vitro astrocytes were used for experiments.

### 4.3. Immunocytochemical Method

In order to detect GFAP and PI3K in cells, we used an immunocytochemical assay. The cells were fixed with 4% paraformaldehyde + 0.25% glutaraldehyde in PBS for 20 min and washed three times with ice-cold PBS for 5 min. Glutaraldehyde was added into the fixative solution to minimize washing of antibodies from cells during permeabilization. To permeabilize cells, we used 0.1% Triton X-100 solution for 15 min. Fixed cells were incubated in 10% donkey serum for 30 min at room temperature to block nonspecific antibody-binding sites. The cells were then incubated with primary antibodies against the investigated proteins for 12 h at 4 °C. The fixed cells were subsequently washed with PBS (3 times for 5 min) and probed with secondary antibodies conjugated with with fluorescent label. We used purified mouse monoclonal anti-GFAP antibody (BioLegend, RRID: AB_2632644), purified rabbit monoclonal antibody to PI3-Kinase p85 alpha ([EPR18702], (ab191606)), donkey polyclonal secondary antibody to rabbit IgG (H+L) (Alexa Fluor-647) (Jackson ImmunoResearch Europe LTD, RRID: AB_2492288), and donkey polyclonal secondary antibody to mouse IgG-H&L (Alexa Fluor-594) (Abcam, RRID: AB_2732073). Dilutions of primary and secondary antibodies were performed according to the manufacturer’s recommendations for immunocytochemical staining. The fluorescence of antibodies was visualized with a Leica TCS inverted confocal microscope SP5 (Leica, Germany). Registration of the secondary antibodies’ fluorescence for the control and experimental groups of cell cultures was carried out at the same microscope setting. Fluorescence analysis was performed in Image J 2002 software (Wayne Rasband, Kensington, MD, USA, RRID: SCR_003070) using the Analyze particles and Time series analyzer plugins.

### 4.4. Fluorescent Ca^2+^ Measurements

To detect the changes in [Ca^2+^]_i_, cell cultures were loaded with Fura-2 (4 µM; 40 min incubation; 37 °C). The cells were stained with the probe dissolved in Hank’s balanced salt solution (HBSS) composed of (mM): 156 NaCl, 3 KCl, 2 MgSO4, 1.25 KH2PO4, 2 CaCl2, 10 glucose, and 10 HEPES, pH 7.4. To measure [Ca^2+^]_i_, we used the system based on a Leica DMI6000B inverted motorized microscope with a HAMAMATSU C9100 high-speed monochrome CCD camera. For excitation and registration of Fura-2 fluorescence, we used the FU-2 filter set (Leica, Germany) with BP340/30 and BP387/15 excitation filters, an FT-410 beam splitter, and a BP510/84 emission filter. Illuminator Leica EL6000 with a high-pressure mercury lamp was used as a source of excitation light. To distinguish neurons and astrocytes, we used short-term applications of 35 mM KCl and 10 µM ATP before the main experiments. This method was described in detail in our previous work [68]. Briefly, KCl induces depolarization of excitable cells, which contain a wide range of voltage-gated cation channels. KCl-induced depolarization promotes the opening of voltage-gated calcium channels in neurons (predominantly L-type channels). The conductivity and density of cation channels in astrocytes are insufficient to evoke high-amplitude Ca^2+^ response to KCl application. All the Ca^2+^ signals are presented as a 340/380 ratio of Fura-2 fluorescence.

### 4.5. Technique for Simulation of Ischemia-like Conditions

Ischemia-like conditions (oxygen-glucose deprivation (OGD)) were obtained by omitting glucose (HBSS medium without glucose) and by displacement of dissolved oxygen with argon in the leak-proof system [16]. The level of oxygen in the medium was measured using a Clark electrode. Oxygen tensions reached values 30–40 mm Hg or less within 20 min after the beginning of displacement. Ischemia-like conditions lasting for 40 min were created by supplying the oxygen-glucose deprivation (OGD)-medium into the chamber with cultured hippocampal cells. Constant argon feed into the experimental chamber was used to prevent the contact of the OGD-medium with the atmospheric air. 

### 4.6. Assessment of Cell Viability and Apoptosis

Propidium iodide (1 µM) was used to evaluate the number of dead cells in the cell cultures before and after OGD. The cells were stained for 5 min with the probes diluted in HBSS and then rinsed with HBSS. Fluorescence of the probes was detected with a Zeiss Axio Observer Z1 inverted fluorescent microscope using Filter Set 20. Cell death induced by OGD was assessed by propidium iodide staining (PI, 1 µM) before and after the exposures in the same microscopic field. Since PI stains both dead astrocytes and neurons, analysis of calcium signals upon 35 mM KCl application before OGD was used to identify the type of cells. Neurons were identified by the fast transient calcium signal upon KCl application, as described previously. Furthermore, we used the Ca^2+^ signals (presence or absence of a global increase in [Ca^2+^]_i_ during OGD) as an additional indicator of cell viability [69].

Hoechst 33342 (2 µM) and propidium iodide (1 µM) were used to evaluate the number of dead cells in the cell cultures before and after OGD. The cells were stained for 5 min with the probes diluted in HBSS and then rinsed with HBSS. Fluorescence of the probes was detected with a Zeiss Axio Observer Z1 inverted fluorescent microscope using Filter Set 01 and Filter Set 20. Discrimination of early and late apoptotic cells was performed according to the previously described method [2,70]. Five different areas of each cell culture were analyzed. Each experimental group consisted of three cell cultures from different passages.

### 4.7. Measurement of Respiration and Lactate Release

To assess the respiration rate and lactate production of astrocytes, oxygen consumption rate (OCR) and extracellular acidification rate (ECAR) were measured using the Seahorse XFp Analyzer (Seahorse Biosciences, Billerica, MA, USA) according to the manufacturer’s guidelines. OCR characterizes cell respiration, and ECAR is an indirect readout of L-lactate production. Rat astrocytes were plated at 10,000 cells/well in 100 μL of basal medium to a Seahorse 8-well miniplate. The cells were allowed to attach and grow to obtain a monolayer at 37 °C under 5% CO_2_. The plates were checked for uniform spreading and cell confluence under the microscope before metabolic analysis. Growth medium was aspirated, and cells were washed with PBS then replaced with Seahorse DMEM (with 5 mM HEPES, without phenol red, glucose, pyruvate, and L-glutamine). A 2.5 µg or 5 µg amount of SeNPs was added to astrocytes immediately before start of analysis; for control astrocytes, PBS was added in wells. OCR and ECAR were measured in basal conditions and after injection of four compounds affecting bioenergetics: D-glucose (10 mM, Sigma-Aldrich), oligomycin (4,5 μM; Sigma-Aldrich), carbonyl cyanide 4-trichloromethoxyphenylhydrazone (CCCP) (10 μM; Sigma-Aldrich), and rotenone/antimycin (2.5/4 μM, respectively; Sigma-Aldrich). Four or three time-point measurements were performed after the addition of each compound (total measurement time: 126 min). Data analysis was performed using XFp Wave software 2.6.1.

### 4.8. Extraction of RNA

MagJET RNA Kit (Thermo Fisher Scientific, Waltham, MA, USA) was used for the extraction of total RNA. The RNA quality was estimated by electrophoresis in the presence of 1 μg/mL ethidium bromide (2% agarose gel in Tris/Borate/EDTA buffer). The concentration of the extracted RNA was determined with a NanoDrop 1000c spectrophotometer. RevertAid H Minus First Strand cDNA Synthesis Kit (Thermo Fisher Scientific, USA) was used for reverse transcription of total RNA.

### 4.9. Real-Time Polymerase Chain Reaction (RT-qPCR)

Each PCR was performed in a 25 μL mixture composed of 5 μL of qPCRmix-HS SYBR (Evrogen, Moscow, Russia), 1 μL (0.2 μM) of the primer solution, 17 μL of water (RNase-free), and 1 μL of cDNA. Dtlite Real-Time PCR System (DNA-technology, Moscow, Russia) was used for amplification. The amplification process consisted of initial 5 min denaturation at 95 °C, 40 cycles of 30 s denaturation at 95 °C, 20 s annealing at 60–62 °C, and a 20 s extension step at 72 °C. The final extension was performed for 10 min at 72 °C. All the sequences were designed with FAST PCR 5.4 and NCBI Primer-BLAST software. The data were analyzed with Dtlite software (DNA-technology, Moscow, Russia). The expression of the studied genes was normalized to gene encoding Glyceraldehyde 3-phosphate dehydrogenase (GAPDH). Data were analyzed using Livak’s method [71].

### 4.10. Preparation and Characterization of Selenium Nanoparticles

The method of obtaining and characterizing selenium nanoparticles was described in detail in our previous work [24]. Briefly, SeNPs were obtained by laser ablation in deionized water. The solid target was placed at the bottom of a cuvette under a thin layer of water. In this state, the solid target was irradiated with a laser beam (λ = 1064 nm; T = 4-200 ns; f = 20 kHz; P = 20 W; Ep = 1 mJ). The laser beam was mixed on the target using a TM 2D galvanomechanical scanner (Ateko, Moscow, Russia). The prepared Se nanoparticles consisted of zero-valent Se and had the same characteristic size. The nanoparticle size was characterized using a DC24000 analytical centrifuge (CPS Instruments, USA). We used monomodal size nanoparticles with an average size of about 100 nm and half-width in the range 70–120 nm (Supplementary Figure S1).

### 4.11. Statistical Analysis

All presented data were obtained from at least three cell cultures from 2 to 3 different passages. All values are given as mean ± standard error (SEM) or as individual cellular signals in experiments. Statistical analyses were performed by paired *t*-test. Differences are significant * *p* < 0.05, ** *p* < 0.01, and *** *p* < 0.001. n/s—data not significant (*p* > 0.05). MS Excel, ImageJ, Origin 2016 (OriginLab, Northampton, MA, USA), and Prism GraphPad 7 (GraphPad Software, RRID: SCR_002798) software were used for data and statistical analysis.

## 5. Conclusions

SeNPs dose-dependently induce the generation of Ca^2+^ signals in astrocytes, various parts of the brain through the activation of the phosphoinositide signaling cascade, and the mobilization of Ca^2+^ ions from the thapsigargin-sensitive ER pool. The generation of Ca^2+^ signals in the astrocytic network involves the mechanisms of vesicular Ca^2+^-dependent ATP secretion through connexin (Cx43) channels, which activates the purinergic receptors of nearby astrocytes. SeNPs protect cortical cells from OGD-induced necrosis through suppression of Ca^2+^ signals, decreased respiration, and increased release of lactate by astrocytes. The neuroprotective effects of SeNPs during ischemia/reoxygenation involve changes in the expression of genes encoding kinases, neurotrophins, and transcription factors. The preliminary 24 h treatment of the neuroglial culture of the mouse cerebral cortex with SeNPs and the subsequent oxygen-glucose deprivation led to the reactivation of astrocytes, mainly of the A2 phenotype, with neuroprotective properties. At the same time, the effect on cells only of SeNPs, or under OGD conditions without preliminary incubation with SeNPs, was not observed.

## Data Availability

The data presented in this study are available on request from the corresponding author.

## Supplementary Materials

The following are available online at https://www.mdpi.com/article/10.3390/ijms222312825/s1.
