# Supplementary material for: The Mechanisms Underlying the Protective Action of Selenium Nanoparticles against Ischemia/Reoxygenation Are Mediated by the Activation of the Ca2+ Signaling System of Astrocytes and Reactive Astrogliosis
Source: Int J Mol Sci. 2021 Nov 26;22(23):12825. doi: 10.3390/ijms222312825 (PMC8657910; doi:10.3390/ijms222312825)
Supplement: Supplementary file 1 [file ijms-22-12825-s001.zip › ijms-1460104-supplementary.pdf]

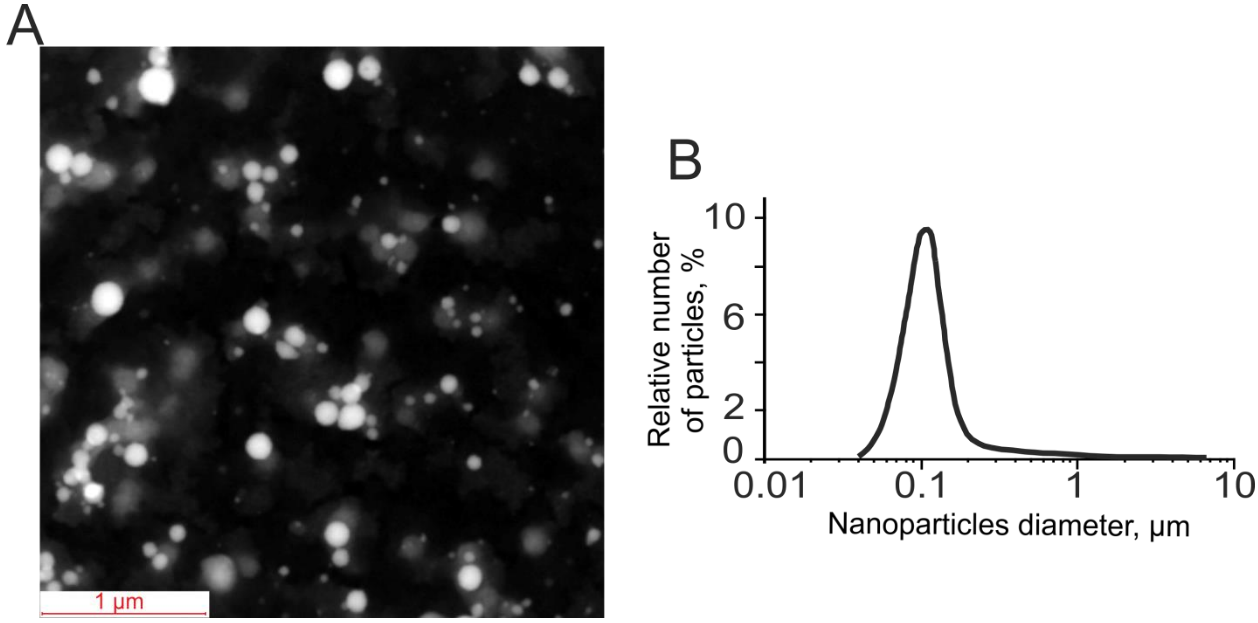

Figure S1. Size distribution and morphology of selenium nanoparticles obtained by laser ablation. **A** – TEM micrograph of selenium nanoparticles. **B** – Selenium nanoparticle size distribution. Data obtained using an analytical disk centrifuge and confirmed by DLS.
